# Association Between Age and Sleep Quality: Findings From a Community Health Survey

**DOI:** 10.1192/j.eurpsy.2025.568

**Published:** 2025-08-26

**Authors:** J. Jeong, J. Hong

**Affiliations:** 1 Psychiatry, St. Vincent’s Hospital The Catholic University of Korea, Suwon; 2 Psychiatry, Iksan Hospital , Iksan, Korea, Republic Of

## Abstract

**Introduction:**

With increase in life expectancy, there is growing interest in quality of life. Sleep is receiving increasing attention among the elderly.

**Objectives:**

This study aimed to investigate the changes in sleep quality with increasing age and the effect of age on the components of the Pittsburgh Sleep Quality Index (PSQI).

**Methods:**

We used data from the Community Health Survey conducted by the Korea Center for Disease Control and Prevention in 2018. A total of 228340 participants in this nationwide survey. Sleep quality was assessed using the PSQI. Adults aged ≥ 19 years were divided into six age groups and one-way analysis of variance (one-way ANOVA) was used to compare the mean values of PSQI of each group. By comparing the scores for each PSQI component in those aged ≥ 65 years and < 65 years, we aimed to reveal the differences in special components according to age group.

**Results:**

In total, 223334 respondents were included in the study. Based on a one-way ANOVA, the PSQI score generally increased with age. Although the average PSQI score of patients in their 40s was lower than that of patients in their 30s, there was no significant difference between the two groups (p = 0.11). When the PSQI component was compared between the population aged over and under 65 years, the population aged ≥ 65 years scored higher in most components. In contrast, daytime dysfunction scored higher in the population aged < 65 years.

**Conclusions:**

Sleep quality tends to decrease with increasing age. Several factors, including physiological changes, underlying physical conditions, and psychosocial factors, may contribute to a decrease in sleep quality with age.

**Disclosure of Interest:**

None Declared

